# Supplementary material for: Social network analysis for the assessment of pig, cattle and buffalo movement in Xayabouli, Lao PDR
Source: PeerJ. 2019 Jan 9;6:e6177. doi: 10.7717/peerj.6177 (PMC6330034; doi:10.7717/peerj.6177)
Supplement: Supplemental Information 1 [file peerj-07-6177-s001.docx]

***Questionnaire for Social Network Analysis*** ***of Pig, Cattle, Goat and Buffalo Traders***

1. Date of interview ________________________________________________________________
2. Interviewee name and phone number _________________________________________________
3. Interviewer name and phone number _________________________________________________
4. Age _________

Gender Male Female

1. Occupation (check all that apply)

Pig farmer (O1) Animal worker (O8) Cattle farmer (O2) Meat seller (O7)

Buffalo farmer (O5) Seller (O6) Animal collector (O3) Slaughter (O4)

Goat farmer (O9) Others________________________

1. Where is your place of business or where do you raise animals?

Village___________________________, Unit___________________________, District___________________________, Province___________________________

1. If you are a collector, animal trader, or animal raiser, please use other questionnaire and answer Topic 9.
2. Type of animal husbandry in your household?

| Tick | Type of animals | Total |
| --- | --- | --- |
| 1 | Cattle |  |
| 2 | Buffalo |  |
| 3 | Pigs |  |
| 4 | Goats |  |

1. Where did you originally get the animals from? (check all that apply)

| No. | Type of animals | Name of owner/seller  (animal’s original source) | Telephone number | Occupation of person involved with animals | Where did animals originally come from (live market, farms, etc.)? | Name of their original source  (Slaughterhouse, market, etc.) | Location of their source (village, district, province, etc.) | How many times per year were animals acquired? | Average number of animals per time | How were the animals transported? |
| --- | --- | --- | --- | --- | --- | --- | --- | --- | --- | --- |
|  | 1 Cattle |  |  | O1 Pig | 1 Farm |  |  |  |  | 1 Pickup |
|  | 2 Buffalo |  |  | O2 Cattle | 2 Market |  |  |  |  | 2 Motorbike |
| 9.1 | 3 Pig |  |  | O3 Collector | 3 Slaughterhouse |  |  |  |  | 3 Truck |
|  | 4 Goat |  |  | O4 Slaughter | 4 Field |  |  |  |  | 4 Walking |
|  |  |  |  | O5 Buffalo | 5 Public land |  |  |  |  | 5 Other |
|  |  |  |  | O6 Seller | 6 Other |  |  |  |  |  |
|  | 1 Cattle |  |  | O1 Pig | 1 Farm |  |  |  |  | 1 Pickup |
|  | 2 Buffalo |  |  | O2 Cattle | 2 Market |  |  |  |  | 2 Motorbike |
| 9.2 | 3 Pig |  |  | O3 Collector | 3 Slaughterhouse |  |  |  |  | 3 Truck |
|  | 4 Goat |  |  | O4 Slaughter | 4 Field |  |  |  |  | 4 Walking |
|  |  |  |  | O5 Buffalo | 5 Public land |  |  |  |  | 5 Other |
|  |  |  |  | O6 Seller | 6 Other |  |  |  |  |  |
|  | 1 Cattle |  |  | O1 Pig | 1 Farm |  |  |  |  | 1 Pickup |
|  | 2 Buffalo |  |  | O2 Cattle | 2 Market |  |  |  |  | 2 Motorbike |
| 9.3 | 3 Pig |  |  | O3 Collector | 3 Slaughterhouse |  |  |  |  | 3 Truck |
|  | 4 Goat |  |  | O4 Slaughter | 4 Field |  |  |  |  | 4 Walking |
|  |  |  |  | O5 Buffalo | 5 Public land |  |  |  |  | 5 Other |
|  |  |  |  | O6 Seller | 6 Other |  |  |  |  |  |
|  |  |  |  |  |  |  |  |  |  |  |
|  | 1 Cattle 2 Buffalo |  |  | O1 Pig  O2 Cattle | 1 Farm  2 Market |  |  |  |  | 1 Pickup  2 Motorbike |
| 9.4 | 3 Pig |  |  | O3 Collector | 3 Slaughterhouse |  |  |  |  | 3 Truck |
|  | 4 Goat |  |  | O4 Slaughter | 4 Field |  |  |  |  | 4 Walking |
|  |  |  |  | O5 Buffalo | 5 Public land |  |  |  |  | 5 Other |
|  |  |  |  | O6 Seller | 6 Other |  |  |  |  |  |
|  | 1 Cattle |  |  | O1 Pig | 1 Farm |  |  |  |  | 1 Pickup |
|  | 2 Buffalo |  |  | O2 Cattle | 2 Market |  |  |  |  | 2 Motorbike |
| 9.5 | 3 Pig |  |  | O3 Collector  O4 Slaughter  O5 Buffalo  O6 Seller | 3 Slaughterhouse |  |  |  |  | 3 Truck |
|  | 4 Goat |  |  |  | 4 Field |  |  |  |  | 4 Walking |
|  |  |  |  |  | 5 Public land |  |  |  |  | 5 Other |
|  |  |  |  |  | 6 Other |  |  |  |  |  |
|  | 1 Cattle |  |  | O1 Pig | 1 Farm |  |  |  |  | 1 Pickup |
|  | 2 Buffalo |  |  | O2 Cattle | 2 Market |  |  |  |  | 2 Motorbike |
| 9.6 | 3 Pig |  |  | O3 Collector | 3 Slaughterhouse |  |  |  |  | 3 Truck |
|  | 4 Goat |  |  | O4 Slaughter | 4 Field |  |  |  |  | 4 Walking |
|  |  |  |  | O5 Buffalo | 5 Public land |  |  |  |  | 5 Other |
|  |  |  |  | O6 Seller | 6 others |  |  |  |  |  |
|  |  |  |  |  |  |  |  |  |  |  |
| 9.7 | 1 Cattle 2 Buffalo 3 Pig  4 Goat |  |  | O1 Pig  O2 Cattle  O3 Collector  O4 Slaughter  O5 Buffalo  O6 Seller | 1 Farm  2 Market  3 Slaughterhouse  4 Field  5 Public land  6 Other |  |  |  |  | 1 Pickup  2 Motorbike  3 Truck  4 Walking  5 Other |
| 9.8 | 1 Cattle  2 Buffalo  3 Pig  4 Goat |  |  | O1 Pig  O2 Cattle  O3 Collector  O4 Slaughter  O5 Buffalo  O6 Seller | 1 Farm  2 Market  3 Slaughterhouse  4 Field  5 Public land  6 Other |  |  |  |  | 1 Pickup  2 Motorbike  3 Truck  4 Walking  5 Other |

1. Where do you do you send animals to? (check all that apply)

| No | Type of animals | Name of owner/buyer  (animal’s destination) | Telephone number | Occupation of person involved with animals | What is animal’s destination?  (Slaughterhouse, market, farmer, etc.) | Name of their destination (Slaughterhouse, market, etc.) | Location of their destination (village, district, province, etc.) | How many times per year are animals sent? | Average number of animals per time | How were the animals transported? |
| --- | --- | --- | --- | --- | --- | --- | --- | --- | --- | --- |
|  | 1 Cattle |  |  | O1 Pig | 1 Farm |  |  |  |  | 1 Pickup |
|  | 2 Buffalo |  |  | O2 Cattle | 2 Market |  |  |  |  | 2 Motorbike |
| 10.1 | 3 Pig |  |  | O3 Collector | 3 Slaughterhouse |  |  |  |  | 3 Truck |
|  | 4 Goat |  |  | O4 Slaughter | 4 Field |  |  |  |  | 4 Walking |
|  |  |  |  | O5 Buffalo | 5 Public land |  |  |  |  | 5 Other |
|  |  |  |  | O6 Seller | 6 Other |  |  |  |  |  |
|  | 1 Cattle |  |  | O1 Pig  O2 Cattle  O3 Collector  O4 Slaughter  O5 Buffalo  O6 Seller | 1 Farm |  |  |  |  | 1 Pickup |
|  | 2 Buffalo |  |  |  | 2 Market |  |  |  |  | 2 Motorbike |
| 10.2 | 3 Pig |  |  |  | 3 Slaughterhouse |  |  |  |  | 3 Truck |
|  | 4 Goat |  |  |  | 4 Field |  |  |  |  | 4 Walking |
|  |  |  |  |  | 5 Public land |  |  |  |  | 5 Other |
|  |  |  |  |  | 6 Other |  |  |  |  |  |
|  | 1 Cattle |  |  | O1 Pig  O2 Cattle  O3 Collector  O4 Slaughter  O5 Buffalo  O6 Seller | 1 Farm |  |  |  |  | 1 Pickup |
|  | 2 Buffalo |  |  |  | 2 Market |  |  |  |  | 2 Motorbike |
| 10.3 | 3 Pig |  |  |  | 3 Slaughterhouse |  |  |  |  | 3 Truck |
|  | 4 Goat |  |  |  | 4 Field |  |  |  |  | 4 Walking |
|  |  |  |  |  | 5 Public land |  |  |  |  | 5 Other |
|  |  |  |  |  | 6 Other |  |  |  |  |  |
|  |  |  |  |  |  |  |  |  |  |  |
| 10.4 | 1 Cattle  2 Buffalo  3 Pig  4 Goat |  |  | O1 Pig  O2 Cattle  O3 Collector  O4 Slaughter  O5 Buffalo  O6 Seller | 1 Farm  2 Market  3 Slaughterhouse  4 Field  5 Public land  6 Other |  |  |  |  | 1 Pickup  2 Motorbike  3 Truck  4 Walking  5 Other |
|  | 1 Cattle |  |  | O1 Pig  O2 Cattle  O3 Collector  O4 Slaughter  O5 Buffalo  O6 Seller | 1 Farm |  |  |  |  | 1 Pickup |
| 10.5 | 2 Buffalo |  |  |  | 2 Market |  |  |  |  | 2 Motorbike |
|  | 3 Pig |  |  |  | 3 Slaughterhouse |  |  |  |  | 3 Truck |
|  | 4 Goat |  |  |  | 4 Field |  |  |  |  | 4 Walking |
|  |  |  |  |  | 5 Public land |  |  |  |  | 5 Other |
|  |  |  |  |  | 6 Other |  |  |  |  |  |
|  | 1 Cattle |  |  | O1 Pig | 1 Farm |  |  |  |  | 1 Pickup |
|  | 2 Buffalo |  |  | O2 Cattle | 2 Market |  |  |  |  | 2 Motorbike |
| 10.6 | 3 Pig |  |  | O3 Collector | 3 Slaughterhouse |  |  |  |  | 3 Truck |
|  | 4 Goat |  |  | O4 Slaughter | 4 Field |  |  |  |  | 4 Walking |
|  |  |  |  | O5 Buffalo | 5 Public land |  |  |  |  | 5 Other |
|  |  |  |  | O6 Seller | 6 Other |  |  |  |  |  |
|  |  |  |  |  |  |  |  |  |  |  |
|  | 1 Cattle |  |  | O1 Pig  O2 Cattle  O3 Collector  O4 Slaughter  O5 Buffalo  O6 Seller | 1 Farm |  |  |  |  | 1 Pickup |
|  | 2 Buffalo |  |  |  | 2 Market |  |  |  |  | 2 Motorbike |
| 10.7 | 3 Pig |  |  |  | 3 Slaughterhouse |  |  |  |  | 3 Truck |
|  | 4 Goat |  |  |  | 4 Field |  |  |  |  | 4 Walking |
|  |  |  |  |  | 5 Public land |  |  |  |  | 5 Other |
|  |  |  |  |  | 6 Other |  |  |  |  |  |
|  | 1 Cattle |  |  | O1 Pig  O2 Cattle  O3 Collector  O4 Slaughter  O5 Buffalo  O6 Seller | 1 Farm |  |  |  |  | 1 Pickup |
|  | 2 Buffalo |  |  |  | 2 Market |  |  |  |  | 2 Motorbike |
| 10.8 | 3 Pig |  |  |  | 3 Slaughterhouse |  |  |  |  | 3 Truck |
|  | 4 Goat |  |  |  | 4 Field |  |  |  |  | 4 Walking |
|  |  |  |  |  | 5 Public land |  |  |  |  | 5 Other |
|  |  |  |  |  | 6 Other |  |  |  |  |  |

1. Is there any place that your animal shares the same habitat with others? If yes, please specify. (check all that apply)

0  No.

1  Yes, the water is at: (please specify)

- - 1. ______________________________________________________________________
    2. ______________________________________________________________________
    3. ______________________________________________________________________
    4. ______________________________________________________________________

2 Yes, the pasture is at: (please specify)

- - 1. ______________________________________________________________________
    2. ______________________________________________________________________
    3. ______________________________________________________________________
    4. ______________________________________________________________________

3 Yes, there are water and pasture at: (please specify)

- - 1. ______________________________________________________________________
    2. ______________________________________________________________________
    3. ______________________________________________________________________

11.3.4 ______________________________________________________________________

1. Did this place ever experienced an FMD outbreak in pig, cattle, or buffalo in the past year?

0 No.

1 Yes, (please specify from water or pasture) ___________________________

Not sure. Animals showed clinical symptoms such as: ___________________________

1. Furthermore, animals have other diseases _________________________________________________

___________________________________________________________________________________

1. Do you usually FMD vaccinate your animals?

0 No

1 Yes. How many times per year? ________________________

1. Where do your animals receive vaccinations from?

1 PAFO/DAFO/Project

2 Veterinary services

3 Other. Please specify. ______________________________________________

1. What cattle or buffalo production system are you using?
2. Free range grazing

2 Partial free areas

3 Semi-free and partial

4 Completely tethered

5 Other. Please specify. ______________________________________________________

1. What pig production system are you using?

1 Backyard

1. Small farm
2. Semi-commercial farm

4 Other. Please specify. ______________________________________________________

1. Characteristics of animal farming

|  | No | Yes |
| --- | --- | --- |
| 18.1 Animals shared with other species? | 0 | 1 |
| 18.2 Animals shared with other species? (many owners) | 0 | 1 |
| 18.3 Have boars? (do not share with other villages) | 0 | 1 |
| 18.4 Is there a person who provides insemination services? | 0 | 1 |
| 18.5 Is there a freelance slaughterer for places raising livestock? | 0 | 1 |
| 18.6 Is there a kitchenware seller for places raising livestock? | 0 | 1 |
| 18.7 Is there an animal trader for places raising livestock? | 0 | 1 |
| 18.8 Is there any places that collect manure for places raising livestock? | 0 | 1 |
| 18.9 Are there vet services/VVW for places raising livestock? | 0 | 1 |

1. Are there any people who come to help raise the animals? Please specify people.

______________________________________________________________________________________________________________________________________________________________________

1. Geography for raising animals (check all that apply)

20.1 Specials land 20.2 Mountain/forest

20.3 Flooding/wetland 20.4 Public land

20.5 Nearby road 20.6 Nearby river

20.7 Nearby market 20.8 Nearby slaughterhouse

20.9 Nearby live animal market 20.10 Rice field

20.11 Nearby weekend market 20.12 Other __________________

1. Do you have any suggestions or comments about other information that the authors of this questionnaire may have forgotten to ask about? Please specify if you are an animal trader, animal farmer or other. _________________________________________________________________________________________________________________________________________________________________________________________________________________________________________________________

(Thank you for your cooperation)
